# Environmental and occupational respiratory diseases – 1051. Intractable sneezing – case report

**DOI:** 10.1186/1939-4551-6-S1-P50

**Published:** 2013-04-23

**Authors:** Vattikonda Sathavahana Chowdary

**Affiliations:** 1 Head & Neck Surgery, Allergy Clinic., Apollo Hospitals, Jubilee Hills, Hyderabad, India

## Background

Intractable Sneezing (IS) is a relatively uncommon disorder. It has been reported in about 45 cases in literature. We report one such case and how it was managed is described.

## Methods

An 11 year old female child was referred to the Allergy clinic in the Otolaryngology department with recurrent spells of sneezing of about 2 ½ months duration. Each spell consisted of more than 200 sneezes in 25 minutes.The child underwent clinical evaluation by primary care physician and ENT specialist. There was no relief of symptoms with the standard treatment.A thorough clinical and psychosocial history was taken from the child and the parents. Clinical examination and relevant investigations to exclude any organic causes for sneezing were done. Child’s psychological assessment was done by the clinical psychologist.

## Results

A final diagnosis of psychogenic Intractable Sneezing (PIS) was made.The psychological history revealed the evidence related to psychological stress. She was not able to cope up with her peer group in the school with respect to academic pressures and expectations because of learning disabilities.A result of the above situation was anxiety. So she was using intractable sneezing as a defense to cope up with stress, so that she could abstain from going to school.The child underwent behavioral and psychotherapy and the parents underwent counseling.There was a complete resolution of symptoms in a period of 4 weeks.

## Conclusions

A review of the literature suggests that 38 out of 45 reported cases of intractable sneezing (i.e. nearly 85%) are supposed to be psychogenic in origin. PIS is supposed to be a manifestation of conversion disorder. It is typically seen in children and adolescents and is more common in females. The treatment is basically, psychotherapy with or without anxiolytic medication. PIS is an unusual disorder, but a timely diagnosis of this condition can avoid unnecessary medical trials, parental anxiety and poor school performance as most of the cases are very young.

